# The Effect of Dietary *Camelina sativa* Oil or Cake in the Diets of Broiler Chickens on Growth Performance, Fatty Acid Profile, and Sensory Quality of Meat

**DOI:** 10.3390/ani9100734

**Published:** 2019-09-27

**Authors:** Sylwia Orczewska-Dudek, Mariusz Pietras

**Affiliations:** Department of Animal Nutrition and Feed Science, National Research Institute of Animal Production, 32-083 Balice, Poland; mariusz.pietras@izoo.krakow.pl

**Keywords:** *Camelina* oil, *Camelina* cake, polyunsaturated fatty acids, growth performance, broiler chicken

## Abstract

**Simple Summary:**

Feeding broiler chickens components rich in polyunsaturated fatty acid (PUFA), especially n-3 family fatty acid (*Camelina* oil or expeller) can be an effective way to improve both animal health and meat quality. The rate of mortality was the lowest in the group fed *Camelina* oil or expeller. Broiler chicken meat enriched with bioactive PUFA n-3 can be an alternative source of these fatty acids in the human diet. Introduction to the broiler diet of 40 g/kg *Camelina* oil, as well as 100 g/kg *camelina* expeller cake, significantly increased PUFA n-3 fatty acid and lowered PUFA n-6/PUFA n-3 fatty acid ratio. Furthermore, meat of chickens fed with *Camelina* oil was characterized by better juiciness.

**Abstract:**

The aim of the present study was to determine the effect of supplementing the diets of broiler chickens with *Camelina sativa* oil or cake as a source of polyunsaturated fatty acids (PUFAs) on their growth performance, fatty acid profile, and sensory quality of meat. The 456 Ross 308 broilers aged 21–42 days were divided into 3 groups with 4 replicates of 38 birds in each. Chickens in the control group I (CTR) were fed a standard grower–finisher feed mixture containing 60 g/kg rapeseed oil. The experimental components, *C. sativa* oil—CSO (group II) or cake—CSC (group III), were included in a diet based on wheat and soybean at 40 and 100 g/kg, respectively. The use of *Camelina* oil and cake as feed components did not have a significant effect on the growth performance of the chickens. Analysis of the fatty acid profile in the lipids of the breast muscles showed that *Camelina* oil and cake reduced the content of monounsaturated fatty acids (*p* < 0.05) but increased the content of n-3 polyunsaturated fatty acids, especially α-linolenic acid (C18:3) (*p* < 0.01). Furthermore, both components reduced the ratio of n-6/n-3 PUFAs in the breast muscles (*p* < 0.01). Sensory analysis revealed that *Camelina* oil had a beneficial effect on meat juiciness, whereas *Camelina* cake slightly worsened the flavor and tastiness of the meat. In conclusion, supplementing the diet of broiler chickens with *Camelina* oil or cake can be an efficient method for modifying the fatty acid profile of the meat lipids in a beneficial way, without any negative impact on the growth performance of the chickens. According to the dietetic recommendations for humans, broiler chicken meat with a higher level of PUFA n-3 can be a good alternative source of these fatty acids in the human diet. Furthermore, *Camelina* oil improved the juiciness of breast meat.

## 1. Introduction

The ratio of n-6/n-3 polyunsaturated fatty acids (PUFAs) in the feed mixtures used for fast-growing broiler chickens plays a significant role in the prevention of metabolic disturbances [1] and heart failure, which is a cause of sudden cardiac death [2,3,4]. Numerous studies have indicated that the main cause of the sudden death of birds is the high content of n-6 PUFA in the feed mixtures. The results showed that the serum and heart muscle of these birds contained increased amounts of arachidonic acid (AA; C20:4) and a reduced total level of n-3 PUFA, especially eicosapentaenoic acid (EPA; C20:5n-3) [3,5,6]. Furthermore, the fatty acid profile of meat lipids is a significant factor determining meat quality. The ratio of n-6/n-3 PUFAs in chicken meat ranges from 7:1 to 15:1, and the breast muscles (*Pectoralis major*) are characterized by the most beneficial proportion [7,8]. The wide variation of this ratio is the result of using feed mixtures based on cereal seeds (corn, barley, wheat, and triticale) and plant oils (sunflower, corn, and soybean), as well as oilseeds characterized by a high content of n-6 linolenic acid [9].

Studies have revealed that the meat of chickens, similar to other monogastric organisms, can be efficiently enriched with n-3 PUFA by using an appropriate diet [10,11]. It was found that the use of feed mixtures supplemented with oils as a source of n-3 PUFA during the second growth phase of broiler chickens modified the fatty acid profile of meat lipids in a beneficial way [12,13]. The introduction of oil rich in α-linolenic acid (ALA) in the feed mixture used for broiler chickens increased the concentration of this acid and its long-chain derivatives, including EPA, DPA, and docosahexaenoic acid (DHA) in the meat lipids, which resulted in a decreased proportion of n-6/n-3 PUFAs [8,12]. Compared to mammals, broiler chickens have a greater ability to convert ALA to long-chain derivatives due to higher activity and wider substrate specificity of elongases responsible for the conversion of DPA to C24:5n-3 and then to DHA [14,15].

According to Haug et al. [8], the inclusion of poultry meat as a potential source of n-3 PUFA in the diet of contemporary populations can contribute to reducing the risk of cardiovascular diseases. In their experiment, the authors modified the fatty acid profile of the leg and breast muscles in broiler chickens by using a feed supplemented with a mixture of rapeseed and flax oil as a source of ALA. People consuming such enriched meat for 4 weeks showed an increased concentration of EPA in the serum. The results of other authors have also indicated that the meat of broiler chickens can be included as a potential source of n-3 PUFA in the human diet [11,16,17].

The oil of *Camelina sativa* is one of the richest known plant sources of ALA of the n-3 group [18,19]. *Camelina sativa* is an oil plant that attracts renewed interest of industry and agriculture after it was replaced in the post-war period by higher yielding rapeseed. The renewed interest in *Camelina sativa* results from a higher demand for fat raw materials necessary for production of biofuels. *Camelina* belongs to the oldest crop plants of the *Brassicacea* family. Soil requirements of this plant are modest; it can grow on poor soils and is resistant to drought and frost [20]. Moreover, it requires a lower fertilization rate than rapeseed and is resistant to insect pests [19,20]. *Camelina* oil is considered valuable mostly due to its nutritional values and chemical composition [19]. In particular, the cold-pressed oil is characterized by a high content of PUFAs and natural antioxidants such as tocopherols (791 mg/kg) [20] that make it exceptionally durable and fit for human consumption for 6 months [18]. It is also distinguished from other oils by a special taste and pleasant clear flavor of medium intensity. *Camelina* oil of a domestic variety has been shown to have high efficacy in modifying the fatty acid profile of meat lipids in broiler chickens [21,22,23]. In addition, it was observed to have a beneficial effect in reducing the ratio of n-6/n-3 PUFAs [22,23]. On the other hand, *Camelina* cake is characterized by high protein content (up to 45%) with a beneficial composition of amino acids [24] and fat content with a high proportion of ALA [19,25]. The energy value of *Camelina* cake in poultry, pig, and cattle was estimated at 8.0, 14.0, and 15.0 MJ ME/kg DW, respectively [24]. However, due to the presence of non-starch polysaccharides and glucosinolates, adding a high percentage of *Camelina* cake in the feed mixture can adversely affect the growth performance of broiler chickens [26,27].

Therefore, the aim of the present study was to determine the effect of *C. sativa* oil or expeller cake included as components in the diets of broiler chickens on the growth performance, fatty acid profile of lipids in the breast muscle (*Pectoralis major*), and sensory quality of meat.

## 2. Materials and Methods

### 2.1. Birds, Housing, and Feeding

The experiment was carried out according to the guidelines of the Ethics Committee for the Use of Animals in Research. No explicit approval of the committee was needed because the birds were only fed different diets (none of them were toxic—the regulation 68/2013 (16 January 2013) of the European Union Commission allowed the use of *C. sativa* seeds and products obtained by their processing, including oil and *Camelina* meal, as a feed component in animal diets) and no invasive procedures were performed on them. A total of 456 Ross 308 broiler chickens (hens and cockerels) were raised in group pens on litter from 1 to 42 days of age under standard housing conditions with free access to feed and water. During the first growth phase (1–21 days of age), the chickens were fed a starter feed mixture which did not contain the tested additives. In the second period of rearing (22–42 days of age), chickens were randomly divided into 3 groups with 4 replicates of 38 birds each. The chickens in the control group CTR (group I) were fed a standard grower diet containing 60 g/kg of rapeseed oil, whereas birds in the experimental groups were fed a feed mixture containing 40 g/kg of cold-pressed oil obtained from the seeds of spring *C. sativa* var. Borowska—CSO (group II) and 20 g/kg of rapeseed oil, or 100 g/kg of expeller cake—CSC obtained from *Camelina* of the same variety and 50 g/kg of rapeseed oil (group III). The composition and nutritional value of the grower feed mixtures used for the broiler chickens are presented in Table 1. Diets were formulated to provide nutrients according to the Polish recommendations for broilers [28] and to contain the same amount of metabolizable energy and crude protein within each set.

### 2.2. Data Collection and Chemical Analyses

The individual body weight of the chickens was determined at 1, 21, and 42 days of age, and the number of dead birds was noted throughout the experiment. Feed intake by the groups was determined for each pen. On the basis of the experimental data collected, the following basic parameters of production were calculated: Body weight gain (BWG), feed conversion ratio (FCR) per kilogram BWG, and mortality of birds. At the end of the experiment, at 42 days of age, 8 birds from each group (4 cocks and 4 cockerels) were slaughtered using a method adapted to their age, species, and body weight. The procedure was carried out in accordance with the Annex IV of European Parliament and Council Directive 2010/63/EU of 22 September 2010 on the protection of animals used for scientific purposes. If no pain or suffering was inflicted during the trial, the regulations allowed sacrificing the experimental birds before sampling. Accordingly, the birds were electrically stunned and then decapitated. The mass of fresh carcasses (after slaughter), as well as mass of cold carcasses (after cooling it for 24 h in temperature of +4 °C for 24 h), were determined. The simplified slaughter analysis of the carcasses was performed after cooling them at +4 °C for 24 h [29]. Samples of the breast muscles were collected for further analysis.

The diets were analyzed for the profile of higher fatty acids (HFA) and the content of tocopherols and tocotrienols. The content of HFA was determined using the modified method by Loor and Herebain [30] based on ISO 12966–2:2011. The fatty acids were separated in the form of methyl esters and determined using a VARIAN 3400 gas chromatograph with a flame ionization detector (250 °C, range = 11; carrier gas: helium, 3 mL/min; gas injection: 0.7 μL) and an RTX™-2330 capillary column (105 m × 0.32 mm, 0.2 μm). Tocopherols and tocotrienols were determined using liquid chromatography, according to the method described by Manz and Philip [31], with a Merck-Hitachi HPLC system equipped with a LiChroCART^®^ 250–4 Superspher^®^ 100 RP-18 cartridge on a 4-µm column and an FL detector (Ex. 295 nm and Em. 350 nm).

The collected samples of the breast muscles (*musculus Pectoralis major*) were analyzed for basic chemical composition, fatty acid profile, sensory parameters, and malonaldehyde content after 90 days of frozen storage (−20 °C). The basic chemical composition of the breast meat was determined according to the AOAC method [32]. The content of fatty acids in meat lipids was determined in the form of methyl esters using gas chromatography according to the procedures validated by the Central Laboratory of the National Research Institute of Animal Production in Aleksandrowice, Poland. Fat was extracted from the samples with a mixture of chloroform and methanol (2:1) according to the modified method of Floch et al. [33], and the extract was evaporated at 65 °C under nitrogen. The residue was saponified with 0.5 NaOH in methanol (80 °C, 20 min), and then esterified with BF in methanol [34] at 80 °C for 10 min, followed by the addition of hexane. After salting out with a saturated NaCl solution, the hexane layer was collected into a chromatographic vial and subjected to gas chromatography using a VARIAN 3400 system with an RTX-2330 capillary column (105 m × 0.32 mm, 0.2 μm; detector range = 11, 250 °C; carrier gas: helium, 3 mL/min), a Varian 8200 CX Autosampler, and Varian Star 4.5 software package for data analysis.

The TBA values in the breast meat were expressed as milligrams of malonaldehyde (MDA) per kilogram of meat. To determine TBA, meat samples were prepared according to a modified version of the method of Salih [35], as modified and described by Pikul [36]. TBA values were measured by the colometric method at the presence of 2-thiobarbituric acid.

Breast meat samples held for sensory analysis were frozen at –20 °C until evaluation. Breast meat was analyzed after cooking to determine the sensory impact of the tested components used on flavor and tastiness quality. The sensory evaluation of meat samples was conducted by eight internal panelists. The day before the analysis, 200 g of each sample was thawed at 4 °C, and cooked individually in a covered container in 400 mL of 0.6% saline, until the temperature inside the meat reached 70 °C. The temperature was measured using a special thermometer. After cooling, the meat samples were evaluated within 10 min. The flavor, juiciness, tenderness, and tastiness of the meat were evaluated based on a 5-point scale, where 5 meant strong appreciation and 1 an extreme dislike, on the basis of the method described earlier by Matuszewska and Baryłko-Pikielna [37].

### 2.3. Statistical Analysis

For evaluating the growth performance during the experimental period of 21–42 days, a total of 162 birds per treatment with 4 replications each were considered. As the other results were analyzed by including 8 replications per treatment, statistical analysis of the obtained indices was performed using a one-way analysis of variance. The significance of differences between the experimental groups was evaluated using the multiple-range Duncan test. The differences were deemed statistically significant at a confidence level of *p* < 0.05. The procedures were carried out using the SAS statistical package (version 9.2), procedure GLM.

## 3. Results

The addition of *Camelina* seed oil or expeller to the grower feed mixture used for broiler chickens influenced its PUFA profile (Table 2).

The use of *C. sativa* oil or expeller cake reduced the content of saturated fatty acids (SFAs) from 19% (found in the feed mixture used for control group) to 16.1% and 11.7%, respectively, in the feed mixture used for experimental groups, and increased the content of n-3 PUFA (from 1.5% to approximately 12%), especially ALA. The supplemented feed mixtures were characterized by a narrow ratio of n-6/n-3 PUFAs amounting from 2.1 to 2.5 compared with the control feed mixture (6.8). The contents of α- tocopherol in the feed were similar in all the groups. Supplementing the diet with *Camelina* oil, and especially cake, increased the content of γ-tocopherol by 20.4% and 110%, respectively, and slightly decreased the level of β-tocopherol (Table 3).

The level of gamma-tocopherol in the feed mixture used for experimental groups was 36.6 (CSO group) and 63.9 mg/kg (CSC group), while that in the feed used for the control group was 30.4 mg/kg. The content of delta-tocopherol in the feed containing *Camelina* oil (4.01 mg/kg) or cake (5.7 mg/kg) was higher compared with the feed used for the control group (3.26 mg/kg) by approximately 23% and 75%, respectively.

Rearing results of broiler chickens are shown in Table 4. At the first period of rearing, there were no significant differences in the body weight of chickens and feed consumption, as well as in feed consumption ratio between the groups. Mortality rate in each group was similar and ranged from 0.91 to 1.29%. The addition of *Camelina* oil or cake to feed mixtures during the second growth phase did not significantly affect the BWG of the experimental groups compared with the control group. Moreover, the feed intake and conversion per kilogram of BWG remained at a similar level in all of the groups. Most birds that died during the second growth phase and the whole experimental period belonged to the CTR group (Table 4).

The addition of *Camelina* oil or cake to the diet did not affect the carcass weight and slaughter yield (Table 5). However, a reduction in the percentage of abdominal fat was noted in the carcasses in the CSO and CSC groups. The carcasses of chickens fed *Camelina* oil (CSO) were characterized by the largest weight and the greatest percentage of breast muscles (*p* < 0.05) and the lowest fat content. The carcasses of chickens in the CSO and CSC groups also showed the lowest percentage of the liver compared with the CTR group. A significant (*p < 0.05*) reduction in proportion of skin with subcutaneous fat was observed in the carcasses of chickens from the CSO group.

The use of *Camelina* oil and cake as grower diet components for broiler chickens did not significantly affect the dry mass and the content of total protein and crude fat in the breast muscles (*Pectoralis major*) (Table 6).

The results of the analysis of PUFAs in the lipids of the breast muscles (*Pectoralis major*) demonstrated that *Camelina* oil and cake caused a highly statistically significant (*p* < 0.01) increase in the content of n-3 PUFA, especially ALA (Table 7).

In addition, a significant increase (*p* < 0.01) in EPA was noted in the CSO group, which was fed with the diet supplemented with 4% *Camelina* oil. In the CSO group, as well as in the CSC group, the content of AA (C20:4) belonging to n-6 PUFA was significantly reduced (*p* < 0.05). The ratio of PUFA/SFA was significantly higher and the ratio of n-6/n-3 PUFAs was significantly reduced (*p* < 0.01) in groups II and III, compared with the control group. In the CSO and CSC groups, the content of monounsaturated fatty acids (MUFAs) was statistically significantly reduced (*p* < 0.01) compared with the CTR group, while the level of erucic acid (C22:1) was significantly increased (*p* < 0.01). In the CSC group receiving the *Camelina* oil-supplemented diet, the SFA content was significantly increased compared to the CTR and CSC groups (*p* < 0.05), mostly due to the increased levels of palmitic acid (C16:0) (*p* < 0.01) and stearic acid (C18:0) (*p* < 0.05). Among the acids of this group, significant increases were observed in AA (C20:0) (*p* < 0.01) and behenic acid (C22:0) (*p* < 0.05). The addition of 4% *Camelina* oil to the diet also significantly increased the content of conjugated linoleic acid (CLA) in the CSO group (*p* < 0.01).

**Figure 1:**
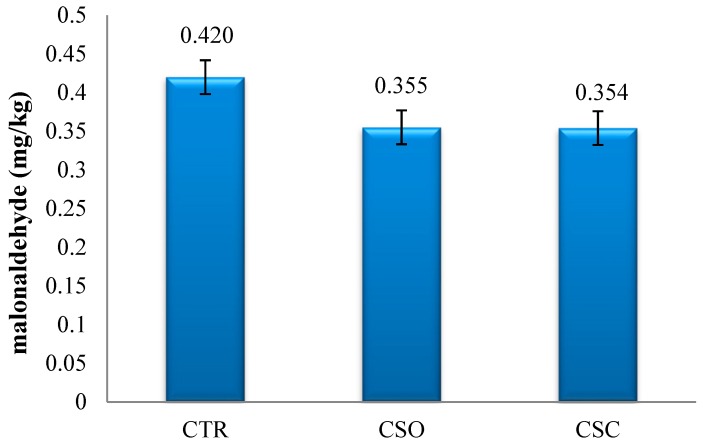
Malonaldehyde content (mg/kg of sample) in the breast muscles (*Pectoralis major*) of broiler chickens fed with rapeseed oil—CTR group, *Camelina* oil—CSO group, and *Camelina* expeller cake—CSC group.

After 3 months of frozen storage (−20 °C), the content of malonaldehyde was found to be reduced (Figure 1) by 19% and 20%, respectively, in the meat of the broiler chickens in the CSO and CSC groups compared with the CTR control group. However, statistical analysis did not confirm the significance of these differences.

Supplementation of the diet of broiler chicken with *Camelina* oil (CSO group) significantly (*p* < 0.05) influenced the juiciness of the cooked meat compared with the control group (Table 8). On the other hand, the meat of the CSC group chickens fed *Camelina* cake-supplemented diet was characterized by an inferior tastiness and flavor.

This section may be divided by subheadings. It should provide a concise and precise description of the experimental results, their interpretation, as well as the experimental conclusions that can be drawn.

## 4. Discussion

The quality and nutritional value of oilseeds, oil pressed from them, and the by-products of oil production depend on their chemical compositions, fatty acid profile, and especially, the contents of antinutrients [26,27,38]. Recent studies have indicated that the oil and cake of *C. sativa* can be used as a source of PUFAs, mostly of the n-3 group, and natural antioxidants such as tocopherols without any impairment of the sensory quality of the poultry products [39,40,41,42]. Antinutrients contained in *Camelina* seeds include mostly glucosinolates, the content of which mainly depends on the variety and environmental conditions that prevail during the plant vegetation [38]. Moreover, the seeds contain crude fiber which can also have a negative impact on the production performance of chickens. Thus, the efficiency of production depends on the choice of *Camelina* variety and the percentage of seeds or by-products of oil pressing in the feed mixtures.

In the present study, the addition of 4% *Camelina* oil to the diet of broiler chickens did not significantly affect their growth performance. Moreover, *Camelina* oil had no effect on the final body weight and FCR. Similar results were shown by Pietras and Orczewska-Dudek [23], who investigated the effect of the addition of 3% and 6% dietary *Camelina* oil to broiler diet. In addition, Jaśkiewicz et al. [21] demonstrated that the addition of *Camelina* oil to broiler diet both during the first (1.43%) and second phase of growth (2.16%) did not adversely affect the production parameters. Analogous results were reported by Jaśkiewicz et al. [22] who used 6.91% *Camelina* oil in the starter diet and 4.07% *Camelina* oil in the grower diet.

The addition of *Camelina* cake to feed mixtures caused a slight reduction in growth rate, and thus led to a lower BWG in chickens. Feed conversion per kilogram of BWG was also found to be slightly increased in this group; however, the differences were not confirmed as significant by statistical analysis. Similar results were obtained by Aziza et al. [42,43] who supplemented the feed with 2.5, 5, and 10% *Camelina* cake. These authors indicated that the addition of *Camelina* cake to the diet of broiler chicken at 10% did not impair the production performance. On the other hand, Pekel et al. [44] demonstrated a negative effect of *Camelina* cake supplement on the production performance of chickens. They found that the addition of 10% *Camelina* cake in the feed mixture used for broiler chickens diminished their growth between 15 and 37 days of age, reducing feed intake, which resulted in a significant reduction of the final body weight. This could probably have resulted from a higher content of glucosinolates in *Camelina* cake used in their experiment, a higher level of fiber, and a limitation of nutrient availability [38].

Valkonen et al. [45] showed that increasing the content of *Camelina* cake (from 0 to 25%) in the feed mixtures used for broiler chickens produced a linear negative effect on the feed consumption, body weight, and feed conversion, but a beneficial effect in lowering the mortality. The authors indicated that the best growth performance was obtained using 5% and 10% *Camelina* cake in the feed. Moreover, the study by Widyaratne [46] demonstrated that when the percentage of *Camelina* cake in the feed mixtures used for broiler chickens was increased from 3 to 15%, the BWG of chickens and feed intake decreased in direct proportion to the increase. *Camelina* cake also had a negative effect on feed conversion (kg/kg BWG). Furthermore, when *Camelina* seeds were added to feed mixture at 30%, a slower growth rate and a low final body weight were observed. It was observed [41] that the addition of 5% and 10% *Camelina* cake to the diets of turkeys led to both growth depression and reduction of feed intake in the birds. These discrepancies between the present study and the above-mentioned studies could probably be due to the use of different varieties of *Camelina* grown under different climatic conditions. This was confirmed by the study of Waraich et al. [20], who indicated that the *Camelina* variety, climatic conditions during vegetation, and fertilization program influenced the contents of fatty acids, vitamins, and glucosinolates in seeds.

The lower percentage of dead birds noted during the second growth phase in groups fed with the diet containing *Camelina* oil or cake was in agreement with the results obtained by Aronen et al. [47]. It can be expected that the beneficial effect of *Camelina* oil is associated with its high content of ALA, which contributes to a reduction in the formation of pro-inflammatory eicosanoids when chickens are fed a diet deficient in ALA but containing high levels of phytosterols. Moreover, it was found that both *Camelina* oil and cake are a rich source of ALA of n-3 group and caused a reduction in the ratio of n-6/n-3 PUFAs in the feed mixtures used for broiler chickens which, according to Chen et al. [48], propitiously increased the level of antioxidant enzymes in the heart muscle and improved the immunological function of the thymus. In addition, Świątkiewicz et al. [49] suggested that n-3 PUFA improved the immunological functions in animals; in particular, they reduced the prevalence of acute and chronic inflammatory response generated towards harmful factors.

The present study demonstrated that the addition of 10% *Camelina* cake significantly reduced the proportion of the breast muscle in the carcasses by 7.5% compared with the control group. However, Aziza et al. [42,43] did not notice such a relationship. These authors concluded that irrespective of the percentage, *Camelina* cake did not have any negative effect on the quality and tissue composition of the chicken carcasses. In our study, it was observed a reduction in the content of abdominal fat in the carcasses of chickens that received the feed mixture supplemented with 4% *Camelina* oil or 10% cake. These results are in accordance with the data obtained earlier in the study by Pietras and Orczewska-Dudek [23], who also observed a reduction in the percentage of abdominal fat and weight of skin with subcutaneous fat in the carcasses of chickens fed with diet containing 6% *Camelina* oil.

On the other hand, Jaśkiewicz et al. [22] did not notice any significant effect of 2.04% *Camelina* oil added in the feed mixture used for chickens on the content of abdominal fat in the carcasses. According to Valkonen et al. [45], the amount of abdominal fat in the carcasses of chickens that were fed the diets supplemented with *Camelina* cake linearly decreased with an increase in the content of *Camelina* cake in the feed (from 5 to 10%). Similar results were reported by Crespo and Esteve-Gracia [13] and Ferrini et al. [50], who revealed that PUFAs reduced the accumulation of abdominal fat in contrast to SFAs and MUFAs. According to Takeuchi et al. [51] and Sanza et al. [52], PUFAs inhibited the synthesis of lipids in the liver and enhanced the processes of thermogenesis. This mechanism explains why PUFAs reduce fat in the abdomen and other parts, and as a consequence, decrease the total content of fat in a carcass [52,53]. In our study, both *Camelina* oil and cake did not affect the content of crude fat in the breast muscles of the broiler chickens. This is in line with the reports of other authors [13,54], who concluded that the source of fat in feed mixtures and their fatty acid profile did not influence the content of crude fat in the meat samples of broiler chickens.

The results of the analysis of HFA in the lipids of the breast muscles (*Pectoralis major*) indicate that both *Camelina* oil and cake significantly reduced the percentage of MUFAs, especially oleic acid, and increased the percentage of PUFAs, mostly of the n-3 group. According to the dietetic recommendations for humans, reducing the ratio of n-6/n-3 PUFAs is desirable because a narrow ratio of n-6/n-3 PUFAs is beneficial for maintaining a proper balance between eicosanoids formed from both groups of fatty acids [55,56]. The obtained results confirmed the data reported by other authors [22,23,42,43]. According to Thacker and Widyaratne [46], the addition of 15% *Camelina* cake induced a statistically significant increase in the content of n-3 PUFA and beneficially narrowed the ratio of n-6/n-3 PUFAs. In addition, Nain et al. [57] indicated that feeding broiler chickens with both a mixture enriched with 24% *Camelina* cake for 28 days and 16% *Camelina* cake for 42 days significantly increased the content of n-3 PUFA in the lipids of the breast and leg muscles, exceeding the content of 300 mg/100 g of meat.

In the present investigation, the lowest level of MUFAs was observed in the breast muscles from chickens that were fed with the mixture supplemented with 4% *Camelina* oil. In addition, the desaturation index SCD-1 (C18:1/C18:0) was significantly lower in this group, which can indicate a reduction in the activity of stearoyl-CoA desaturase which catalyzes the synthesis of MUFAs in the liver [58]. Moreover, Paton and Natambi [59] and Green et al. [60] confirmed the role of this enzyme as an inhibitor of the synthesis of MUFAs from SFAs, especially of the transformation of stearic acid into oleic acid and palmitic acid into palmitoleic acid. In the present study, the content of EPA was found to be statistically significantly increased in the experimental groups. The increase in EPA resulted from the elevated content of ALA, a precursor of long-chain fatty acids, which was confirmed by the studies Azcon et al. [61] and Jiang et al. [62]. It was found from the experiment that the addition of *Camelina* oil or cake to broiler diet significantly modified the fatty acid profile of the lipids of the breast muscle (*Pectoralis major*), leading to a significant increase in ALA (C18:3n-3).

Other authors [11] also observed that an oil rich in PUFAs influenced the lipid metabolism, leading to a greater accumulation of ALA in tissue lipids and reduction of SFA. As explained earlier, the increase of SFAs in the lipids of breast muscles (*Pectoralis major*) of broiler chickens fed with a mixture enriched with 4% *Camelina* oil could be caused by the suppression of the transformation of SFAs to MUFAs due to the diminished activity of an enzyme participating in these reactions. It is supposed that the highest increase in CLA also observed in this group additionally contributed to the reduction in the activity of SCD-1, which significantly decreased the content of SFAs in the lipids of the breast muscle. The group that received the feed mixture supplemented with *Camelina* cake showed a significantly increased content of linoleic acid (C18:2 n-6). LA is biologically converted into AA, the level of which was found declined in the lipids of the breast muscle (*Pectoralis major*) of chickens fed with *Camelina* oil or cake. Betti et al. [63] also noted in their study that the increase in n-3 PUFA resulted in a reduction of AA in the phospholipids of the breast muscle.

The results of several studies have demonstrated that the meat of animals containing more PUFAs is more susceptible to oxidative processes [63], which has a negative impact on its organoleptic characteristics and shelf life [11]. Supplementation of α-tocopherol to the chicken diet increases its content in body tissues and limits the oxidation of fat in the breast muscles (*Pectoralis major*) [42,43,64]. It was also confirmed that malondialdehyde (MDA) content in the breast muscles (*Pectoralis major*) of broiler chickens in the experimental groups, measured after 3-month frozen storage, was lower by 6% compared with the control group. The high content of natural antioxidants such as tocopherols and tocotrienols in *Camelina* oil and cake increased tocopherol content in the feed, which resulted in its increase in cell membranes, thus slowing down the oxidation of the lipids in the breast muscle (*Pectoralis major*). Moreover, *Camelina* oil was found to contain high levels of phytosterols and phenolic compounds that also contribute to the limitation of PUFA oxidation.

The obtained results agree with those reported by Aziza et al. [42] who discovered that *Camelina* cake added to feed mixture at 10% efficiently restricted the oxidation of fatty acids and improved the oxidative stability of meat lipids. In the present study, supplementation of the feed mixture with 4% *Camelina* oil did not impair the organoleptic quality of the cooked meat, and even significantly improved its juiciness. The obtained results were consistent with the earlier observations of Pietrsa and Orczewska-Dudek [23]. However, these authors did not note the effect of 3% and 6% *Camelina* oil on the juiciness of breast muscle (*Pectoralis major*) in chicken. The current study showed that the addition of 10% *Camelina* cake to the feed mixture used for broiler chickens had a less favorable effect on the sensory quality by worsening the flavor and tastiness of the meat. In contrast, a beneficial effect of 5% *Camelina* cake added as a supplement to the feed mixture used for broiler chickens on the tenderness and juiciness of the meat was documented [41]. Such an effect was not observed when the content of *Camelina* cake was increased to 10%, which was also noticed in presented study. In addition, Valkonen et al. [45] indicated that *Camelina* cake had a favorable effect on the sensory properties of the breast muscles.

## 5. Conclusions

On the basis of the obtained results, it can be concluded that the addition of *Camelina* oil or expeller to the diet of broiler chickens can be an efficient method for modifying the fatty acid profile of the meat lipids in a way that is beneficial according to the dietetic recommendations for humans, without compromising the growth performance of the birds.

A high content of tocopherols in *Camelina* oil and *Camelina* meal slows down the oxidative processes of breast meat lipids, which is reflected in a lower content of malondialdehyde.

Additionally, *Camelina* oil had a beneficial effect on meat juiciness, whereas *Camelina* cake slightly worsened the flavor and tastiness of the meat.

*Camelina* expeller cake can be a cheaper alternative source of polyunsaturated fatty acids, as well as natural antioxidants, but the level of *Camelina* expeller used in broiler chicken diet should be more thoroughly investigated in future.

## Figures and Tables

**Table 1:** Composition and the calculated nutrient contents of grower–finisher feed mixtures (g/kg).

Components (g/kg)	Group		
	CTR	CSO	CSC
Maize, ground	480.0	480.0	500.0
Wheat, ground	104.6	104.6	49.5
Soybean meal (46% protein)	315.0	315.0	260.0
Rapeseed oil	60.0	20.0	50.0
*Camelina* oil	–	40.0	–
*Camelina* cake	–	–	100.0
Ground limestone	11.5	11.5	12.5
Phosphate 2-Ca	17.0	17.0	16.0
NaCl	3.5	3.5	3.5
DL-Methionine	2.1	2.1	1.8
L-Lysine HCl	1.3	1.3	1.7
Vitamin–mineral premix (0.5%) *	5.0	5.0	5.0
Metabolic energy (MJ)	13	13	13
Total protein (g)	200.0	200.0	200.0
Lysine (g)	11.5	11.5	11.5
Methionine (g)	5.2	5.2	5.2
Ca (g)	9.2	9.2	9.2
P available (g)	4.0	4.0	4.0

* Vitamin–mineral premix provided the following (per kg of feed mixture): Retinyl acetate—10,000 IU; cholecalciferol—2000 IU; tocopherol—20 mg; menadione sodium bisulfite—2.0 mg; thiamine—1.5 mg; riboflavin—5 mg; pyridoxine—3 mg; cyanocobalamin—0.02 mg; Ca-pantothenate—12 mg; folic acid—1 mg; biotin—1 mg; niacin—25 mg; choline chloride—400 mg; manganese—100 mg; iodine—0.8 mg; zinc—65 mg; selenium—0.2 mg; and copper—8 mg. Abbreviations: CTR, control group; CSO, *C. sativa* oil group; CSC, *C. sativa* cake group.

**Table 2:** Fatty acid profile of the feed mixtures used for broiler chickens (% of the sum of fatty acids).

Item	Group		
	CTR	CSO	CSC
C16:0	13.764	12.270	9.062
C16:1	0.265	0.199	0.176
C18:0	2.990	2.832	2.095
C18:1	66.191	45.169	42.387
C18:2	9.074	23.655	31.440
γC18:3	0.113	0.247	0.171
C20:0	0.567	0.595	0.445
C18:3	1.353	11.593	12.648
C20:4	0.043	0.494	0.379
C22:1	0.444	1.368	0.788
SFA	19.208	16.141	11.781
MUFA	66.900	46.736	43.351
PUFA	33.891	37.122	44.868
PUFA n-6	9.230	24.397	31.989
PUFA n-3	1.353	11.593	12.648
MUFA/SFA	3.483	2.895	3.680
PUFA/SFA	0.723	2.300	3.808
PUFA n-6/PUFA n-3	6.824	2.104	2.529

**Table 3:** Contents of natural antioxidants in the feed mixtures used for broiler chickens (mg/kg of feed).

Parameter	Group		
	CTR	CSO	CSC
α-Tocopherol	40.01	39.42	39.48
β-Tocopherol	2.67	3.03	2.31
γ-Tocopherol	30.4	36.6	63.9
δ-Tocopherol	3.26	4.01	5.7
α-Tocotrienol	2.44	3.03	3.08
β-Tocotrienol	7.5	8.18	4.82
γ-Tocotrienol	1.79	1.86	3.72
δ-Tocotrienol	0.36	0.35	0.46

**Table 4:** Production parameters of broiler chickens. Abbreviations: BWG, body weight gain.

Variable	Age (d)	Groups			SEM	*p*-Value
		CTR	CSO	CSC		
BWG (g/bird)	1–22	666	671	679	5.17	0.210
	22–42	1856	1834	1734	26.35	0.117
	1–42	2522	2505	2413	24.35	0.509
Feed intake (g/bird)	1–22	934	957	955	7.95	0.288
	22–42	3075	3025	2959	27.41	0.654
	1–42	4019	3937	3977	23.44	0.684
Feed conversion ratio (kg/kg BWG)	1–21	1.40	1.43	1.34	0.02	0.208
	22–42	1.66	1.62	1.75	0.03	0.393
	1–42	1.59	1.57	1.65	0.02	0.259
Percentage of dead and culled birds (%)	1–22	1.28	1.29	0.91	-	-
	22–42	1.31	0.65	0.56	-	-
	1–42	2.60	1.31	1.74	-	-

**Table 5:** Slaughter analysis of the carcasses of broiler chickens.

Item	Group			SEM	*p*-Value
	CTR	CSO	CSC		
Fresh carcass weight (g)	1865.00	1887.50	1798.75	44.06	0.615
Cold carcass weight (g)	1793.75	1816.25	1725.00	38.05	0.574
Carcass yield (%)	71.79	72.53	72.74	1.85	0.481
Percentage of muscles (%)					
-Breast	28.25 ^a^	28.69 ^a^	26.13 ^b^	2.03	0.020
-Leg	21.29	21.71	21.52	1.73	0.259
Percentage of the liver (%)	2.57	2.48	2.48	0.22	0.888
Percentage of abdominal fat (%)	1.67	1.58	1.52	0.03	0.777
Weight of the skin with subcutaneous fat (%)	6.10 ^a^	5.88 ^b^	5.91 ^a^	0.38	0.759

a, b—the mean values in a row marked with different letters differ statistically significantly at *p* < 0.05.

**Table 6:** Results of the chemical analysis (%) of the breast muscles (*Pectoralis major*).

Item	Group			SEM	*p*-Value
	CTR	CSO	CSC		
Dry mass	25.72	25.25	25.90	0.10	0.449
Total protein	23.89	23.47	23.81	0.13	0.521
Crude fat	1.06	1.10	1.15	0.08	0.183

**Table 7:** Fatty acid profile of the lipids of the breast muscles (*Pectoralis major*) (% of the sum of acids).

Item	Group			SEM	*p*-Value
	CTR	CSO	CSC		
C16:0	19.97 ^A,B^	21.42 ^A^	19.59 ^B^	1.282	0.0001
C18:0	8.24 ^a,b^	8.63 ^a^	7.56 ^b^	0.885	0.045
C16:1	1.57	1.81	1.57	0.140	0.367
C18:1	41.24 ^A^	34.58 ^C^	37.95 ^B^	3.210	0.0001
C18:2	17.45 ^B^	17.61 ^A,B^	18.94 ^A^	0.928	0.009
γC18:3	0.88	0.94	0.94	0.004	0.793
C20:0	0.15 ^B^	0.21 ^A^	0.21 ^A^	0.021	0.006
C18:3	3.57 ^B^	8.07 ^A^	7.26 ^A^	0.954	0.0001
C22:0	0.13 ^b^	0.29 ^a^	0.24 ^a,b^	0.017	0.029
C20:4	3.88 ^a^	3.04 ^b^	2.91 ^b^	0.793	0.035
C22:1	0.08 ^B^	0.14 ^A^	0.14 ^A^	0.002	0.007
EPA	0.62 ^B^	1.00 ^A^	0.71 ^B^	0.031	0.0001
DHA	1.03	1.02	0.98	0.139	0.952
SFA	29.84 ^a,b^	31.79 ^a^	28.68 ^b^	2.163	0.029
MUFA	42.89 ^A^	36.53 ^C^	39.66 ^B^	2.031	0.0001
PUFA	27.27 ^B^	31.68 ^A^	31.65 ^A^	1.643	0.0001
PUFA n-6	21.43	20.75	21.94	1.416	0.155
PUFA n-3	5.21 ^C^	10.09 ^A^	8.94 ^B^	0.577	0.0001
PUFA/SFA	0.92 ^B^	1.00 ^A,B^	1.11 ^A^	1.448	0.006
PUFAs n-6/n-3	4.12 ^A^	2.08 ^C^	2.46 ^B^	0.369	0.0001
CLA	0.63 ^B^	0.86 ^A^	0.77 ^A,B^	0.010	0.0009
C18:1/C18:0	5.00 ^a^	4.00 ^b^	5.02 ^a^	0.098	0.028

a, b—the mean values in a row marked with different letters differ statistically significantly at *p* < 0.05; A, B, C—the mean values in a row marked with different letters differ statistically significantly at *p* < 0.01.

**Table 8:** Results of the sensory analysis of the breast muscles (*Pectoralis major*) of broiler chickens (according to a 5-point scale: 5—the highest score; 1—the lowest score).

Parameter	Group			SEM	*p*-Value
	CTR	CSO	CSC		
Flavor	4.40	4.35	4.05	1.850	0.679
Juiciness	4.05 ^b^	4.62 ^a^	4.25 ^a,b^	1.550	0.029
Tenderness	4.20	4.35	4.26	0.126	0.735
Tastiness	4.15 ^a,b^	4.20 ^a^	3.88 ^b^	0.948	0.043

a, b—the mean values in a row marked with different letters differ statistically significantly at *p* < 0.05.
